# Anabolic steroids-induced delirium: A case report

**DOI:** 10.1097/MD.0000000000021639

**Published:** 2020-08-14

**Authors:** Mohamed Adil Shah Khoodoruth, Adeel Ahmad Khan

**Affiliations:** a Chief Psychiatry Resident, Department of Psychiatry; b Department of Internal Medicine, Hamad Medical Corporation, Qatar.

**Keywords:** anabolic steroids, case report, delirium, neuropsychiatric

## Abstract

**Introduction:**

Anabolic steroids are commonly used by athletes, body builders, and young adults to improve muscle strength. Deleterious effects of anabolic steroids on physical health are well-established. Psychiatric aspects are of particular importance and include psychosis, delirium, mania, depression, and aggression. We describe the case of a young gentleman who was managed as a case of androgenic steroid induced delirium.

**Patient concern:**

A 33-year-old gentleman presented with increased aggression, hostility, and destructive impulses. He was a regular user of testosterone propionate, testosterone cyprionate and trenbolone acetate up to 200 mg daily in injectable form. His mental status examination showed labile effect, flight of ideas and persecutory delusions. Physical examination was positive for atrophic testes. Laboratory results showed a decreased plasma testosterone level of 9.59 nmol/l (10.4–37.4 nmol/l). Sex Hormone Binding Globulin was 23.8 nmol/l (18.3–54.1 nmol/l) and bioavailable testosterone was 5.110 nmol/l (4.36–14.30 nmol/l).

**Diagnosis:**

He was diagnosed as a case of anabolic steroids induced delirium.

**Interventions and outcome:**

Patient was treated with regular haloperidol and quetiapine after which his sensorium, speech and behavior improved. He was discharged on haloperidol 7.5 mg and quetiapine 700 mg daily.

**Conclusion:**

The purpose of this case report is to emphasize on the neuropsychiatric effects and management of anabolic steroids manifested by delirium, increased aggression, hostility, and destructive impulses.

## 1 Introduction

Androgen anabolic steroids (AAS) are frequently used by athletes to improve muscle strength and increase endurance due to their effects on testosterone receptors.^[1]^ However, surveys have shown that up to 80% of anabolic steroids use is by nonathletes including body-builders and young adults.^[2]^ A variety of formulations of anabolic steroids are available. Oral formulations include methyltestosterone, oxandrolone, and stanozolol. With passage of time, users progress to injectable forms of anabolic steroids like nandrolone decanoate and testosterone propionate. Sometime, these steroids are used in doses much higher than the recommended levels.^[3]^ Deleterious effects of using anabolic steroids on physical health are well-established and include high blood pressure, gynecomastia, atrophic testes, edema, acne, and rapid weight gain. However, psychiatric aspects of the use of these steroids are of particular importance and include psychosis, delirium, mania, depression, and aggression among many others.^[3–5]^ We report a case highlighting neuropsychiatric effects of AAS use in a young male. This study was approved by the ethics committee and Institutional Review Board of Hamad Medical Corporation (MRC-04-19-475), and the written informed consent was obtained from the patient.

## 2 Case report

A 33-year-old gentleman, working as a school counsellor, with no family history of mental illness, previously diagnosed as bipolar disorder in 2018, was brought to the emergency department of a general hospital after his colleagues called the Emergency Medical Services when they found him in a confused state in his apartment. In the emergency department, he presented with altered level of consciousness and displayed disorganized and agitated behavior in terms of entering a female cubicle and trying to take another patient's mobile phone, damaging an intravenous drip stand, taking a hook from the wall and damaging the tap of an oxygen supply, which was managed by physical and chemical restraints. His manager reported that he was absent from work for three days and that he found anabolic steroids in his apartment. He was then transferred to the psychiatric inpatient unit for further management, with a provisional diagnosis of substance-induced confusion.

At the beginning of his admission, his mental state examination was significant for labile affect, irrelevant speech, derailment, flight of ideas, and persecutory delusions. He was on high risk one-to-one observations precautions and nursing team have reported disorganized behavior in terms of eating cigarettes, tried to burn his genitals with cigarettes, jumping over the nursing station, unable to dress himself, tried to use the female nursing staff washroom, talkativeness, and disturbed sleeping pattern, in addition to altered level of sensorium throughout the day. He also mistook the female occupational therapist for his mother and tried to kiss her feet. Otherwise, he did not display hallucinatory behavior, grandiose delusions, or elated mood. He was vitally stable and he had a BMI of 29. Physical examination was unremarkable except for testicular atrophy. He also reported taking tadalafil 20 mg for erectile dysfunction.

Past psychiatric history is significant for 1 similar episode in 2018 for which patient was admitted in a psychiatric hospital under the impression of bipolar disorder and was discharged on sodium valproate 2 g daily, olanzapine 20 mg daily, haloperidol 5 mg twice daily, and procyclidine 5 mg BID. He took the medications for 3 months and stopped them because of sedation and weight gain. Collateral history obtained from parents were negative for manic symptoms.

Substance use history is positive for anabolic steroids. He took 5 courses of injectable testosterone propionate and cyprionate, and trenbolone acetate from the age of 21 for bodybuilding purposes. Last had the injectable in May 2019. He described 1 course as 100 to 200 mg daily for 90 days. One month prior this admission he used to take metandienone tablets 20 mg daily.

**Figure 1:**
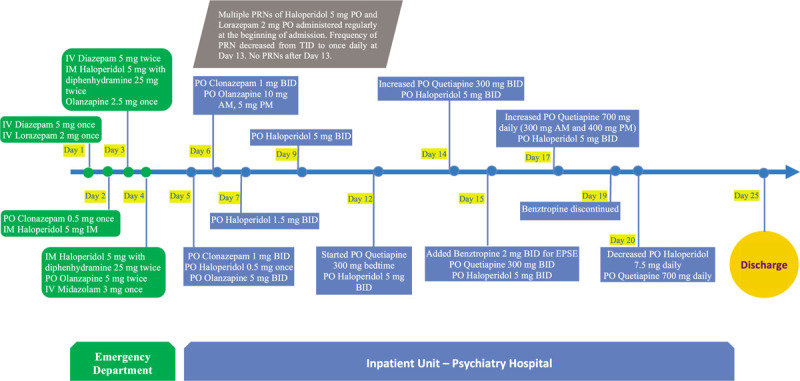
Timeline chart of pharmacological management. The patient stayed in the emergency department from day 1 to 4. On day 5 he was admitted to the psychiatry inpatient unit. He was then discharged on day 25. IV-Intravenous; IM-Intramuscular; PO-Per os (oral route); PRN-as needed; AM-before midday; PM-after midday; BID-twice daily; TID-thrice a day.

Complete blood count, comprehensive metabolic panel, lumbar puncture, computed tomography of the brain were unremarkable. Urine toxicology screen was negative for amphetamines, cannabis, cocaine, and opiate. HIV and syphilis screening were negative. Endocrinology work-up is illustrated in Table 1. He was managed as a case of anabolic steroids induced delirium. Treatment comprised mainly antipsychotics, although at the start of his presentation in the emergency department he necessitated multiple as needed parenteral benzodiazepines and antipsychotics. He was discharged on Haloperidol 7.5 mg and Quetiapine 700 mg daily and left to his home country. A timeline chart of pharmacological management is shown in Figure 1.

**Table 1:**
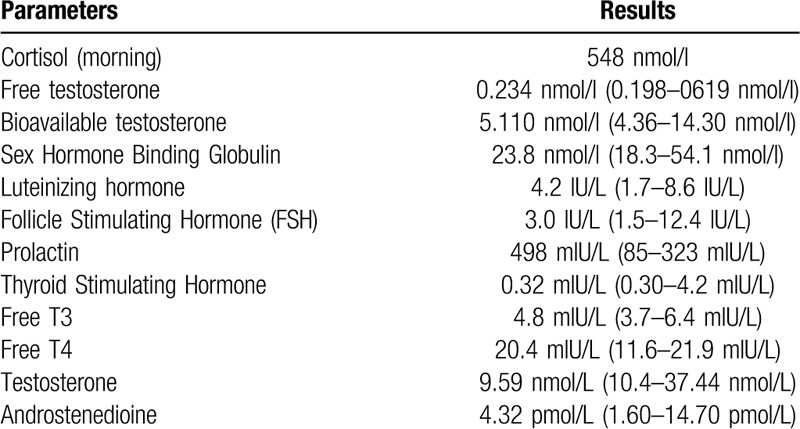
Endocrinology work-up.

## 3 Discussion

One of the cardinal aspects of this case was the management of the aggressive and disruptive behavior. Numerous studies have linked AAS to aggressive behavior.^[6,7]^ Initial animal studies have reported that long-term exposure to high doses of testosterone heightened level of aggression in rats.^[8]^ One probable pathophysiology could be that high aggression is associated to decreased serotonin (5-HT) neurotransmission as evidenced by decreased in both 5-HT and 5-HT metabolite, 5-HIAA, in the hippocampus of adult rats exposed to testosterone propionate.^[9]^ In addition, the aggressive behavior was decreased by treatment with selective agonists of 5-HT1A, 5-HT1B, and 5-HT2A / 2C receptors.^[9]^ Likewise, Ambar and Chiavegatto have reported reduced 5-HT1B mRNA levels in the hippocampus, hypothalamus, amygdala, and prefrontal cortex of nandrolone-treated mice suggesting that the serotonergic tone in these brain areas has a key role for AAS-induced aggression.^[10]^

Another important, albeit less reported and less studied effect of anabolic steroids use is confusion and delirium. In a double-blind, placebo-controlled crossover trial to study neuropsychiatric effects of anabolic steroids, confusion and increased distractibility was also observed, in addition to violence, aggressive behavior and mood related symptoms, with high-dose methyltestosterone (240 mg/d) administration.^[11]^ Cases of delirium following therapeutic use of corticosteroids have been reported as well.^[12]^ The possible mechanisms might be the same as described for AAS-induced aggression and mania.^[3,4]^

As regard to the past psychiatric history of bipolar disorder with our patient, we were doubtful that he indeed suffered from bipolar disorder. We were unable to obtain a medical report of his admission in 2018 as he was in another country at that time. However, collateral history from parents were negative for manic symptoms. Besides, the patient presented with delirium-type of clinical picture together with disorganized behavior rather than a manic type. At best we speculate that his past episode was possibly an AAS-induced manic state in light of a review by Pope et al which reports at least 5% of AAS users will experience AAS dose-dependent manic or hypomanic episodes.^[4]^ There are a few case reports highlighting the occurrence of manic features in context of AAS. For example, Papazisis et al reported the case of a 25-year-old male body builder, with history of depression, who developed mania and psychosis shortly after increasing his AAS dose to >2 g daily, which unfortunately resulted in suicide secondary to AAS discontinuation and ultimately withdrawal-related depression.^[13]^ Of note, the healthy male produces 2.5 to 11 mg of testosterone daily, and the therapeutic dose for treatment for muscle wasting in HIV is approximately 100 to 150 mg daily.^[14,15]^ On the other hand Weiss et al described a case of a 28-year-old gentleman, known to have bipolar disorder, with acquired immunodeficiency syndrome (AIDS) who developed mania after starting the prescribed testosterone patch therapy (2–6 mg daily) 1 month earlier, thus indicating a lower threshold to AAS-induced mania in certain populations. Franey and Espiridion have highlighted the probable increase in susceptibility to develop mania in context of heavy cannabis usage in their case report of a 30-year-old male, when actually previous randomized clinical trials investigating psychiatric effects of AAS have excluded participants with a history of drug abuse.^[1]^

## 4 Patient's perspective

Patient improved significantly after receiving treatment in terms of his orientation and behavior. He was fully oriented by the time he was discharged. He was informed about his diagnosis and that anabolic steroids use was the potential causative factor of his condition. He understood and vowed to abstain from using anabolic steroids use in future.

## 5 Conclusion

The purpose of this case report is to emphasize on the neuropsychiatric effects and management of anabolic steroids manifested by delirium, increased aggression and hostility, and destructive impulses. Physicians and psychiatrists alike should be aware of the dimensions of use of AAS and the complex connection between neuropsychiatric and behavioral presentation. Further studies that will shed light on AAS-induced delirium, especially in the clinical setting, are necessary.

## Author contributions

Mohamed Adil Shah Khoodoruth conceived the topic idea, identified and managed the case. He wrote the case presentation, discussion and conducted literature review. Adeel Ahmad Khan conducted literature review, contributed to writing introduction and discussion. In addition, he critically reviewed the manuscript.
